# Pandora’s box

**DOI:** 10.1192/bji.2025.10

**Published:** 2025-05

## Brain wash is vital

We know sleep is essential for human beings and other animals. Sleep is a time not only for our brains to rest but also for them to have a good clean-up in preparation for the next day; this is vital to our brain health and well-being. Our brains are busy in the awake state, keeping us going physically and mentally, and our neurons and other brain cells are working hard. This vigorous metabolic activity accumulates by-products, chemical waste that needs to be cleared. Amyloid and tau associated with dementia are part of the undesirable waste, and failure in brain cleansing may lead to degenerative disorders. For its cleansing function, the brain depends on the glymphatic system, similar to the way the body depends on its lymphatic system to remove undesirable substances. The cerebrospinal fluid (CSF) plays the part of the lymph, flowing along cerebral blood vessels and seeping through tiny spaces to carry out its brain-washing activity. This CSF flow is not as free as that of the lymph, and what drives its flow during sleep has not been clear.

A study in mice shed some light on this process using a method called ‘flow fibre photometry’ that enabled blood and CSF dynamics to be recorded during rapid eye movement (REM) and non-REM sleep and also wakefulness. The researchers labelled blood using liver-secreted fluorescent-tagged albumin and tagged CSF with fluorescent tracer. Using simultaneous recordings of noradrenaline, blood and CSF, as well as electroencephalogram and electromyogram activity, they discovered an ‘intricate and tightly linked relationship between brain states and fluid dynamics’. The locus coeruleus induced noradrenaline oscillations that were the key drivers of slow vasomotor activity, and enabled the CSF to flow into the brain for glymphatic clearance during sleep. Unravelling this process offers a better understanding of the relationship between vascular dysfunction and glymphatic inefficiency, which may contribute to the development of degenerative brain disorders.

Notably, the researchers also found that the Z hypnotic zolpidem, which acts by enhancing GABAergic activity, impaired noradrenaline-driven oscillations, resulting in reduced glymphatic in-flow. This mouse-based study adds to the need for caution in prescribing such hypnotics long-term, in addition to their dependence potential.

**Hauglund NI, Andersen M, Tokararska K, Radovanovic T, Kjaerby C, Sørensen FL,** et al. Norepinephrine-mediated slow vasomotion drives glymphatic clearance during sleep. *Cell* 2025; **188**: 605–22.

## Blood–brain barrier and ageing

Flushing with the glymphatic system is not enough to keep our brains clean and healthy. The blood–brain barrier is a protective system that prevents harmful substances circulating in our vascular system from entering our brains. The brain endothelial layer, the glycocalyx layer, which consists of a sugar-rich mesh of proteoglycans, glycoproteins and glycolipids, is key to the protective function of the blood–brain barrier. Unfortunately, ageing can impair this protective effect.

In a study in mice, researchers aimed to characterise structural brain changes related to ageing, using transmission electron microscopy and staining with lanthanum nitrate. This enabled visualisation of the brain endothelial glycocalyx layers in young and old mice. They found a decline in brain endothelial *O*-glycosylation, which was associated with increased blood–brain barrier permeability and susceptibility to cerebral bleeding, resulting in impaired cognitive functioning. However, not all is lost; they also found that using adeno-associated viruses to restore core 1 mucin-type *O*-glycans in the brain endothelium improved functioning and resulted in reduced neuroinflammation and improved cognitive function in the aged mice.

**Shi SM, Suh RJ, Shon DJ, Garcia FJ, Buff JK, Atkins M,** et al. Glycocalyx dysregulation impairs blood–brain barrier in ageing and disease. *Nature* [Epub ahead of print] 26 Feb 2025. Available from: https://doi.org/10.1038/s41586-025-08589-9.

## No gadgets for healthier sleep

Sleep problems are widespread across the world, raising concern globally and prompting a search for possible causes. The ‘sleep restriction epidemic hypothesis’ considers technologies such as electronic devices and work demands in industrial societies to be responsible for the reduction in sleep duration.

A recent study aiming to test the sleep restriction epidemic hypothesis examined 54 population-level sleep studies using polysomnography and actigraphy. Actigraphy, a non-invasive method that uses a wristwatch-like device to measure activity, has enabled field study of sleep, having been validated against polysomnography. In contrast to the position of the sleep restriction epidemic hypothesis, the researchers showed that populations in industrial societies actually experienced the longest and most efficient sleep. They also tested the hypothesis of circadian mismatch in industrial societies. This hypothesis suggests that regulated environments with poor chrono hygiene disrupt the alignment of circadian rhythms in industrial societies. The researchers’ findings were in support of the circadian mismatch hypothesis, in that sleepers in non-industrial societies had the best circadian function, indicating that more natural environments with less exposure to artificial light and technology are healthier for us. Circadian rhythms are important to us as they affect not only sleep but also other essential functions such as blood pressure, heart rate, body temperature and hormone secretion.

**Samson DR, Mc Kinnoon L.** Are humans facing a sleep epidemic or enlightenment? Large-scale, industrial societies exhibit long, efficient sleep yet weak circadian function. *Proc Biol Sci* 2025; **292**: 20242319.

## Are we more ‘early birds’ than ‘night owls’?

Our mental health, sense of well-being and mood are known to vary with season and age, as well as during the course of the day. A recent study examined whether there is diurnal variability in the way people experience and report their mental health state, focusing on depression, anxiety, well-being and loneliness in relation to the time of day. The authors used data from 49 218 adults from the University College London Covid-19 Social Study that provided repeat measures from participants over a period of 2 years (March 2020 to March 2022, with 18.5 observations per person), which they analysed using linear mixed-effects models.

They found that in general, people felt best on waking up in the morning and worst at about midnight. They also noted an association with the day of the week and particularly the season, with participants feeling much better in the summer. Unfortunately, as one might expect, feelings of loneliness did not change through the day, or with the day of the week.

You may wonder though, weren’t there any ‘night owls’ among the participants?

**Bu F, Bone JK, Fancourt D.** Will things feel better in the morning? A time-of-day analysis of mental health and wellbeing from nearly 1 million observations. *BMJ Ment Health* 2025; **28**: e301418.

## Cannabis and the brain revisited

As rules have relaxed in many parts of the world, cannabis use has increased globally. There has been more interest in harder drugs, and research into the effects of cannabis has been decreasing. Should we relax about its effects on the brain, given its widespread use?

Researchers in the USA examined the question of the effects of lifetime history of heavy cannabis use and also recent use on brain activation with respect to a range of brain functions in a large sample of young US adults. They established cannabis dependence using the DSM-IV criteria, and used data from the Human Connectome Project (August 2012 and 2015) for young adults (22–36 years) for whom magnetic resonance imaging (MRI) scan, urine toxicology and cannabis use information was available. History of heavy cannabis use was assessed using the semi-structured assessment for the genetics of alcoholism, and participants were grouped as heavy lifetime cannabis users (more than 1000 uses; 8.8% of the total), moderate users (10–999 uses; 17.8%) and non-users (fewer than 10 uses; the largest group at 73.4%). Seventy-six per cent of the participants were White, 13.7% were Black and 6.3% were Asian. All subjects provided urine samples on the day of the MRI scan to assess recent use. Seven tasks were administered during a functional MRI session to assess working memory, reward, emotion, language, motor function, relational assessment and theory of mind. Using a linear mixed-effects regression model, with one model per task, the authors examined the association of lifetime cannabis and recent cannabis use with the mean brain activation value.

Their results showed that heavy lifetime users had lower activation of working memory networks in the anterior insula, medial prefrontal cortex and dorsolateral prefrontal cortex. Recent cannabis use was associated with poorer performance and lower brain activation in working memory and motor tasks. These findings suggest probable long-lasting effects on brain function, at least as far as working memory is concerned. So let’s not relax so much about the ill effects of cannabis.

**Gowin JL, Ellingson JM, Karoly HC, Manza P, Ross JM, Sloan ME,** et al. Brain function outcomes of recent and lifetime cannabis use. *JAMA Netw Open* 2025; **8**(1): e2457069.

